# Selective Recovery of Molybdenum over Nickel and Cobalt from Simulated Secondary Sources Using Bifunctional Ionic Liquid [TOA][Cy272]

**DOI:** 10.3390/ma18163826

**Published:** 2025-08-15

**Authors:** Roshanak Adavodi, Adriana Zuffranieri, Pietro Romano, Soroush Rahmati, Francesco Vegliò

**Affiliations:** Department of Industrial and Information Engineering and Economics (DIIIE), Engineering Headquarters of Roio, University of L’Aquila, 67100 L’Aquila, Italy; roshanak.adavodijolfaee@graduate.univaq.it (R.A.); adriana.zuffranieri@student.univaq.it (A.Z.); pietro.romano@univaq.it (P.R.); francesco.veglio@univaq.it (F.V.)

**Keywords:** ionic liquid, metal extraction, molybdenum, cobalt, nickel, selectivity, recovery, hydrodesulfurization catalysts

## Abstract

The growing demand for ultra-low sulfur fuels has intensified interest in recovering strategic metals from the large volumes of hazardous hydrodesulfurization catalysts that are discarded yearly. This work evaluates a task-specific ionic liquid, tri-n-octylammonium bis(2-,4-,4-trimethylpentyl)-phosphinate [TOA][Cy272], synthesized by the acid–base neutralization of tri-n-octylamine and Cyanex 272. FT-IR spectroscopy confirmed complete proton transfer and the formation of a stable ion pair. Liquid–liquid extraction tests were conducted with synthetic Co–Ni–Mo solutions (0.1–2.5 g/L each), a varying ionic liquid concentration (10–50 vol%), pH (1.5–12.5), and organic/aqueous ratio (1:1). At 35 vol% of ionic liquid and pH 2, the extraction efficiency for Mo reached 94%, with separation factors βMo/Ni = 12 and βMo/Co = 7.5; Co and Ni uptake remained ≤15%. Selectivity decreased at higher metal loadings because of ionic liquid saturation, and an excessive ionic liquid amount (>35%) offered no benefit, owing to viscosity-limited mass transfer. Stripping studies showed that 1 M NH_4_OH stripped about 95% Mo, while leaving Co and Ni in the organic phase; conversely, 2 M HCl removed 92–98% of Co and Ni, but <5% Mo. Overall Mo recovery of about 95% was obtained by a two-step extraction/stripping scheme. The results demonstrate that [TOA][Cy272] combines the cation exchange capability of quaternary ammonium ILs with the strong chelating affinity of organophosphinic acids, delivering rapid, selective, and regenerable separation of Mo from mixed-metal leachates and wastewater streams.

## 1. Introduction

Hydrodesulfurization (HDS) is a well-established catalytic process, extensively employed in petroleum refineries to remove sulfur from petroleum fractions, such as gasoline, diesel, and jet fuels. The combustion of organosulfur compounds present in crude oil feedstocks is a major contributor to SOx emissions, which are harmful air pollutants [1]. In response to increasingly stringent environmental regulations, which mandate that the sulfur content in commercial on-road gas oils should be below 10 ppm, hydrotreatment processes have become essential in modern refineries to ensure cleaner fuel production [2].

HDS catalysts typically consist of molybdenum disulfide (MoS_2_), promoted by cobalt or nickel sulfides, and supported on porous alumina [3]. These active catalytic sites are formed through the sulfidation of metal oxides, with Co and Ni acting as promoters at the MoS_2_ slab edges to enhance the catalytic activity [4]. These catalysts facilitate the cleavage of carbon–sulfur bonds in organosulfur molecules and convert sulfur into hydrogen sulfide (H_2_S) through hydrogenation reactions. However, catalyst deactivation is a common challenge, often caused by coke deposition, contamination by metals or ashes, and the interaction of the catalyst with acidic gases, such as H_2_S, SO_2_, and HCl [5]. Furthermore, the regeneration of spent catalysts is not always economically viable or technically feasible, due to irreversible structural degradation during operation [6].

Globally, the petroleum industry generates approximately 150–170 million tons of spent catalysts annually [5]. These materials are categorized as hazardous waste, primarily due to the presence of heavy metals, such as Ni, V, Co, Mo, and Al, and other toxic compounds that pose serious risks to the environment through potential soil, water, and air contamination [2,7,8]. Regulatory bodies, including Environmental Protection Agencies (EPAs), prohibit the disposal of such waste without adequate metal removal and treatment [9]. Therefore, the recovery of valuable metals from spent catalysts does not only align with environmental regulations, but also represents an economic opportunity [10].

Owing to their high metal content (often surpassing that of primary ores), spent catalysts, which typically contain 4–12 wt% Mo, 15–30 wt% Al, and up to 4 wt% Co or Ni, are considered valuable secondary resources [11,12]. Accordingly, developing efficient and environmentally friendly recovery methods is critical for resource conservation and pollution mitigation [13].

Hydrometallurgical processing is increasingly favored over pyrometallurgical techniques for metal recovery due to its lower energy requirements, reduced greenhouse gas emissions, and enhanced selectivity [6]. Pre-treatment steps, such as oxidation, are commonly applied to improve the leaching efficiency, using acid or base leaching agents [2]. The resulting pregnant leach solution (PLS) contains dissolved metals like Mo, Ni, and Co, which are targeted for recovery through the use of various separation techniques. Among the many approaches explored, including precipitation [14], ion exchange [15], membrane separation [9], adsorption [16], and solvent extraction [17], solvent extraction stands out for its higher efficiency and selectivity [7].

A wide range of extractants has been studied, including organophosphorus acids [18] and amine-based compounds, with extractant selection being largely dictated by the leaching conditions and PLS composition [15]. In recent years, due to their unique physicochemical properties, ionic liquids have emerged as promising alternatives to conventional molecular extractants for use in hydrometallurgical processes. These include high chemical and thermal stability, which enables their use under demanding conditions, such as high acid concentrations and elevated temperatures. ILs also possess low vapor pressure, even at high temperatures, making them environmentally friendly and non-volatile [19,20]. In addition, they are non-flammable and exhibit excellent solvation ability for various metal species, which enhances their performance as extraction media.

A notable subclass of ILs is bifunctional ionic liquids, wherein both the cation and anion contribute actively to the metal extraction process [21]. In contrast to traditional extractants that typically rely on either a single neutral molecule or a single ionic component, bifunctional ILs enable simultaneous interaction with metal species via both ionic components [22]. This dual functionality can lead to enhanced extraction efficiency and selectivity, often through synergistic interactions between the two ionic components. These ILs are highly tunable, enabling tailored metal affinity and solubility through the structural modification of the cation or anion. This flexibility often results in improved selectivity and distribution ratios, reducing the number of separation steps required in complex metal recovery processes [23].

Compared to conventional extractants, such as phosphinic acids (e.g., Cyanex 272) and quaternary ammonium salts (e.g., TOA), bifunctional ionic liquids offer several advantages, including higher chemical stability, improved solubility, lower volatility, and reduced issues, such as third-phase formation and emulsion generation. They can also achieve greater extraction efficiency and selectivity, due to the synergistic interaction between their cationic and anionic components [24]. Additionally, incorporating functional groups from traditional extractants into the ionic liquid structure enhances recyclability and provides a more tunable and robust platform for selective metal recovery [25].

Several studies have demonstrated the selective extraction of Mo, Ni, and Co using ILs. Trihexyl(tetradecyl)phosphonium bis(2,4,4-trimethylpentyl)phosphinate achieved a cobalt-to-nickel separation factor (selectivity coefficient) of 1097 when applied to a HCl-leached solution, with over 99% cobalt extracted at an IL concentration of 0.8 mol/L, pH 5.0, and 60 °C, confirming the high selectivity and efficiency of IL-based systems [26]. In another study, Mo was effectively separated from a sulfate-based Co–Mo/Al_2_O_3_ spent catalyst leachate, using two synthesized bifunctional ILs (R4ND and R4NCY), with R4NCY exhibiting superior selectivity at pH 2.0 and achieving more than 99.9% Mo recovery under optimized conditions [15].

The performance of two ionic liquids, ALI-IL and CYP-IL, based on ammonium (Aliquat 336) and phosphonium (Cyphos EH) cations, with a DEHPA-derived anion, was investigated for their potential to extract vanadium and molybdenum from spent catalysts. The phosphonium-based IL demonstrated superior efficiency, achieving 91% recovery for Mo and 82.5% for V, attributed to its larger size and lower charge density, which enhanced the metal ion interactions [27]. Similarly, Cyphos 104, another phosphonium-based ionic liquid, has shown exceptional selectivity for molybdenum extraction at pH 2, achieving over 99% recovery, by forming stable complexes with Mo species. This enabled efficient solvent extraction and stripping to recover high-purity MoO_3_ [28]. The extracted metals from both processes were subsequently precipitated using NH_4_Cl and BaCl_2_, highlighting the potential of phosphonium-based ionic liquids in regard to sustainable metal recovery from spent catalysts. It is worth mentioning that Protic ionic liquids, such as [TOA][Cy272], prepared via straightforward Brønsted acid–base neutralization reactions from readily available and well-known precursors, offer a more cost-effective alternative to aprotic ionic liquids. Their synthesis avoids complex multi-step procedures, the need for high-purity precursors, and extensive purification processes, resulting in lower production costs and a more environmentally sustainable profile. This simplicity in the preparation process enhances their practicality for large-scale industrial applications [29,30].

Although the application of some ILs has been studied for extracting Mo from aqueous solutions, limited research has addressed its selective separation and recovery in the presence of barrier ions, such as Co and Ni, commonly found in hydrometallurgical streams from spent catalyst recycling. In this study, a bifunctional ionic liquid, [TOA][Cy272], synthesized via a simple neutralization reaction between tri-n-octylamine (TOA) and Cyanex 272, was investigated for its potential use in the selective recovery of molybdenum from Co–Ni–Mo solutions, simulating secondary resources, such as spent hydrodesulfurization catalysts. The formation of [TOA][Cy272] was confirmed using FT-IR analysis, and its extraction performance was systematically evaluated in comparison with its precursor components. Key parameters, including the IL concentration (10–50 vol%), pH (acidic to neutral), and metal concentrations in the aqueous phase (0.1–2.5 g/L), were studied in detail. The ionic liquid demonstrated high selectivity toward molybdate anions under acidic conditions, enabling efficient and selective extraction. Additionally, the system enables straightforward and selective stripping of molybdenum, using simple acidic and alkaline stripping agents. The IL’s facile synthesis, rapid extraction kinetics, high selectivity, and operational simplicity underscore its potential as a sustainable and industrially viable option for metal recovery from complex secondary sources. This work introduces a novel extraction strategy based on a bifunctional ionic liquid platform, bridging a critical gap in the selective extraction of molybdenum using ionic liquids.

## 2. Materials and Methods

### 2.1. Materials

To prepare the aqueous stock solution, cobalt (II) acetate tetrahydrate (>98%, Sigma-Aldrich, St. Louis, MO, USA), nickel (II) acetate tetrahydrate (98%, Sigma-Aldrich, St. Louis, MO, USA), and sodium molybdate dihydrate (>99.5%, Merck KGaA, Darmstadt, Germany) were dissolved in distilled water.

The bifunctional ionic liquid was synthesized using trioctylamine (TOA), as the cationic component, and Cyanex 272 (bis(2,4,4-trimethylpentyl)phosphinic acid (CAS 83411-55-0)), as the anionic component. All of the solvent extraction experiments used kerosene (Carlo Erba, Italy) as the diluent.

Ammonium hydroxide (36.6%, VWR, Radnor, PA, USA) and hydrochloric acid (25%, Carlo Erba) were used to prepare the stripping solutions. All the chemicals were of analytical grade and were used as received without further purification.

### 2.2. Synthesis of Bifunctional Ionic Liquid

**Figure 1:**
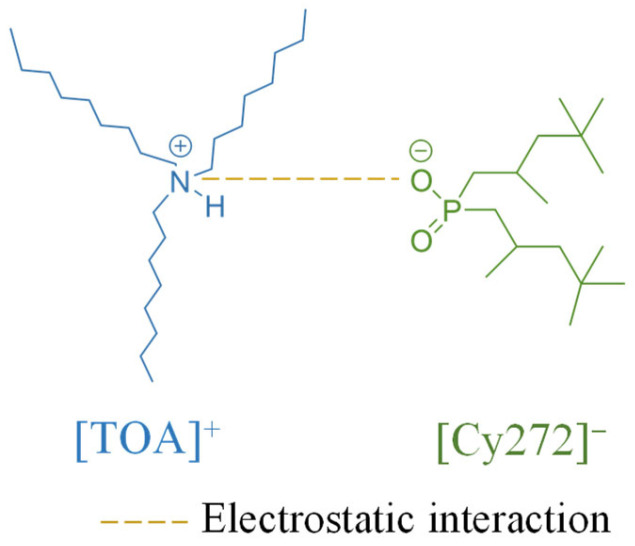
The chemical structure of the synthesized [TOA][Cy272] IL.

The ionic liquid [TOA][Cy272] was synthesized via a neutralization reaction between TOA and Cyanex 272. During a typical procedure, 0.1 mol of TOA was combined with 0.1 mol (an equimolar amount) of Cyanex 272 in a 250 mL Erlenmeyer flask. The mixture was stirred magnetically at 200 rpm, with the temperature initially maintained at 25 °C and gradually increased to 60 °C. The reaction was continued for 12 h to ensure complete formation of the ionic liquid. The chemical structure of the synthesized ionic liquid is shown in Figure 1. No water removal step was required, as the synthesis proceeded via a direct acid–base neutralization process in the absence of aqueous media and without water formation.

### 2.3. Extraction and Stripping Experiments

A series of solvent extraction and stripping experiments was carried out to evaluate the performance of the synthesized [TOA][Cy272] IL for the selective extraction of Mo, Co, and Ni. The effects of key parameters, including metal concentrations in the aqueous phase (100, 1000, and 2500 mg/L for Mo, Co, and Ni), IL concentrations in the organic phase (10, 20, 35, and 50 vol%), the aqueous-to-organic phase ratio (A/O, 0.5,1, 1.5, 2.0 *v*/*v*), and the initial pH of the aqueous feed (1.5, 4.5, and 7.5), were systematically investigated.

In each experiment, the pH values of the aqueous solutions were adjusted and monitored using a Thermo Scientific pH meter (Waltham, MA, USA). The desired pH was obtained by adding small volumes of diluted HCl solution to the aqueous phase, before contact with the organic phase. Then, 25 mL of the organic phase (containing the specified concentration of [TOA][Cy272] dissolved in kerosene) came into contact with 25 mL of the aqueous solution in a glass flask. The phases were mixed using an orbital shaker, set at 250 rpm, for 30 min, at room temperature. This contact time was sufficient to reach equilibrium, as confirmed by preliminary time-dependent experiments. A contact time of 30 min was selected based on preliminary trials, which indicated that equilibrium was effectively reached under the applied conditions, ensuring a reliable comparison of the extraction performance.

After mixing, the two phases were separated using a separation funnel, and the raffinate and loaded organic phase were collected. The extraction efficiency (E), distribution ratio (D), and co-extraction coefficient (β) were calculated using the following equations:

(1) $$E_{i}=100\;*\;\frac{C_{i,o}\times V_{o}}{C_{i,a}^{0}\times V_{a}}$$

(2) $$D_{i}=\frac{C_{i,O}}{C_{i,\;a}}$$

(3) $$\beta_{i/j}=\frac{D_{i}}{D_{j}}$$

where $C_{i,a}^{0}$, $C_{i,a}^{0}$, and $C_{i,o}$ denote the concentrations of metal *i* in the initial aqueous solution, the raffinate, and the organic phase, respectively. $V_{o}$ and $V_{o}$ refer to the volumes of the aqueous and organic phases. The concentrations of metals in the initial aqueous phase and the raffinate solution were measured using inductively coupled plasma optical emission spectrometry (ICP-OES, Agilent 5100, Santa Clara, CA, USA). The concentrations of metals in the organic phase were determined by the mass balance.

For the stripping step, the loaded organic phase obtained from the extraction experiment using 1000 mg/L concentrations of Mo, Ni, and Co (at pH 4.5, with an organic phase containing 35% [TOA][Cy272] and 65% kerosene), came into contact with an equal volume of stripping solution in a glass flask. Two stripping agents were evaluated, namely 2 M HCl (acidic) and 1 M NH_4_OH (alkaline), with the aim of identifying the most efficient reagent for use in the selective stripping stage. The two phases were mixed using an orbital shaker at 250 rpm, for 30 min, at room temperature. After phase disengagement using a separation funnel, the stripped aqueous phase was collected and analyzed using ICP-OES (Agilent 5100) to determine the concentration of each metal. The stripping efficiency (Si) was calculated using the following equation:

(4) $$S_{i}=100\;*\;\frac{C_{i,s}\times V_{s}}{C_{i,o}\times V_{o}}$$

where $C_{i,s}$ and $V_{s}$ represent the metal concentration and the volume of the stripping solution, respectively.

The chemical structures of the synthesized [TOA][Cy272] IL and its precursor components were characterized by Fourier-transform infrared spectroscopy (FT-IR, Thermo Nicolet Nexus 670, Madison, WI, USA). The FT-IR spectra were recorded at room temperature in the range of 4000–500 cm^−1^ for pure liquid samples.

## 3. Results

### 3.1. Confirmation of [TOA][Cy272] IL Formation

**Figure 2:**
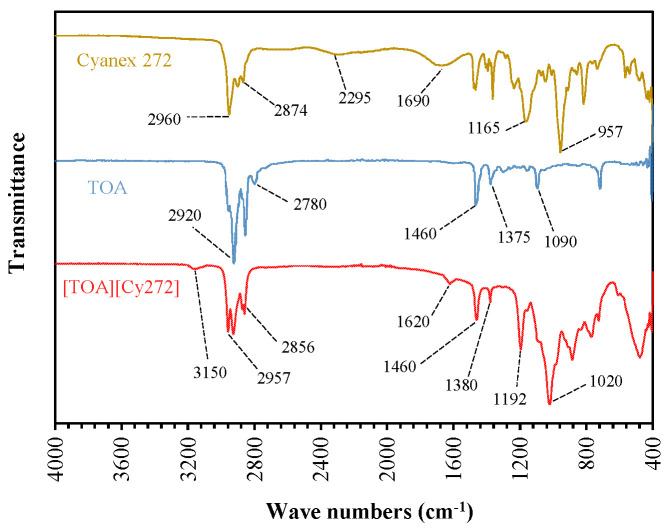
FT-IR spectra of TOA, Cyanex 272, and [TOA][Cy272] IL.

FT-IR spectral analysis was employed to verify the successful synthesis of the ionic liquid [TOA][Cy272] by comparing the spectrum of the final product with those of its starting materials, tri-n-octylamine (TOA) and Cyanex 272 (Figure 2). Key functional groups, including phosphoryl (P=O), hydroxyl (PO–H), and amine (N–H), were analyzed to confirm the formation of the ionic liquid through an acid–base interaction between TOA and Cyanex 272.

In the FT-IR spectrum of Cyanex 272, absorption bands observed at 2960 and 2874 cm^−1^ correspond to the asymmetric and symmetric stretching vibrations of aliphatic CH_2_ groups, respectively [31]. A strong absorption band at 1165 cm^−1^ is assigned to the phosphoryl (P=O) stretching vibration [32]. Although slightly shifted, this band is retained in the spectrum of the synthesized ionic liquid, indicating that the P=O environment is preserved, but is affected by the altered electronic environment following ionic bond formation.

The broad O–H stretching band centered around 2295 cm^−1^ and the deformation vibration at 1690 cm^−1^ in the Cyanex 272 spectrum are indicative of free acidic hydroxyl groups. These bands are absent in the FT-IR spectrum of the ionic liquid, confirming the deprotonation of the acidic group and the formation of the corresponding anionic species ([Cy272]^−^). In addition, the P–OH stretching band, typically observed near 957 cm^−1^ [33], remains present, but is slightly shifted in the IL spectrum, suggesting the retention of the phosphate framework, with minor structural perturbations.

TOA exhibits a characteristic C–H stretching vibration of the alkyl -CH_2_- and -CH_3_ groups in the 2780–2920 cm^−1^ region. The bands at 1465 cm^−1^ (-CH_2_ scissoring) and 1375 cm^−1^ (-CH_3_ umbrella bending) correspond to bending vibrations of the alkyl chains. A distinct absorption near the 1020 cm^−1^ range is assigned to the C–N stretching vibration [34,35]. As expected for a tertiary amine, free TOA lacks N–H stretching features. However, after the reaction with Cyanex 272, new absorption bands appear in the IL spectrum at approximately 3150 cm^−1^ and 1620 cm^−1^. These bands are attributed to N–H stretching and bending vibrations, respectively, and are characteristic of the formation of a protonated amine group ([TOAH]^+^) [36].

Overall, the observed spectral changes, particularly the disappearance of O–H bands and the emergence of new N–H bands, provide strong evidence for the successful synthesis of the ionic liquid [TOA][Cy272] via proton transfer and ion pair formation [32,37].

### 3.2. Assessment of Extraction Efficiency

The extraction performance of the synthesized [TOA][Cy272] IL was assessed through a series of liquid–liquid extraction experiments to investigate its efficiency and selectivity for recovering Co, Ni, and Mo from aqueous solutions under varying conditions.

To systematically evaluate the influence of key operational parameters on metal recovery, controlled experiments were conducted by varying the initial metal concentrations in the aqueous phase, the extractant’s type and concentration, and the solution’s initial pH. These variables are known to significantly impact both the distribution behavior of metal ions and the efficiency of phase separation in solvent extraction systems.

In the following subsections, the effect of each parameter on extraction and separation performance is discussed in detail, providing insights into the underlying mechanisms of metal ion transfer and the selective behavior of [TOA][Cy272].

#### 3.2.1. [TOA][Cy272] vs. Precursors

To evaluate the separation efficiency of the synthesized [TOA][Cy272] IL and benchmark it against its precursors (e.g., TOA and Cyanex 272), three extraction experiments were performed using 35% extractant in kerosene, an A/O phase ratio of 1, and 1000 ppm of each metal at room temperature (Table 1).

The results revealed markedly different extraction behaviors: (1) TOA showed negligible extraction efficiency for Co, Ni, and Mo (approximately 0.0%); (2) Cyanex 272 extracted the metals in the order Co > Mo > Ni, with separation factors βCo/Ni and βCo/Mo of 477.4 and 10.4, respectively, indicating strong selectivity toward Co over both Ni and Mo. The selectivity toward Mo was relatively poor (βMo/Co = 0.10), confirming Cyanex 272’s preference for Co; and (3) [TOA][Cy272] IL extracted all three metals to a moderate extent, with a clear preference for Mo. Torkaman et al. [38] reported that TOA is capable of extracting cobalt from highly acidic chloride media (~6 M HCl). Moreover, Cyanex 272 is a well-established extractant for the selective separation of Co over Ni [39,40,41].

The extraction efficiencies of [TOA][Cy272] IL for Mo, Co, and Ni were 71.8%, 62.6%, and 49.8%, respectively. The corresponding selectivity factors were a βMo/Ni of 2.58 and a βMo/Co of 1.53, indicating the preferential extraction of Mo over both Co and Ni. These results demonstrate that the IL reverses the inherent selectivity trend of Cyanex 272, favoring Mo as the primary extracted metal, while enabling moderate co-extraction of Co and Ni.

It should be noted that bifunctional ionic liquids, such as [TOA][Cy272], extract metals through the combined action of both their cationic and anionic components. Both parts can actively engage in complexation with metal ions or their associated species, thereby enhancing the efficiency of the extraction process [42,43].

In addition, although the Mo/Ni selectivity (βMo/Ni) of the IL is lower than that of Cyanex 272 under identical conditions, subsequent results presented in Section 3.2 demonstrate that [TOA][Cy272] can achieve high selectivity for Mo over both Ni and Co under optimized extraction conditions, reinforcing its potential as a selective system for molybdenum recovery from base-metal mixtures.

These findings suggest that [TOA][Cy272] is an effective extractant for molybdenum recovery from mixed-metal solutions. Therefore, in the following sub-sections, the influence of key parameters on extraction efficiency and Mo selectivity is systematically investigated.

#### 3.2.2. Effect of Metal Concentration

The effect of the metal concentration in the aqueous phase was studied over the range from 100 to 2500 mg/L for Co, Mo, and Ni. This range was selected to reflect concentrations typically encountered in leach solutions derived from secondary sources or industrial wastewater.

**Figure 3:**
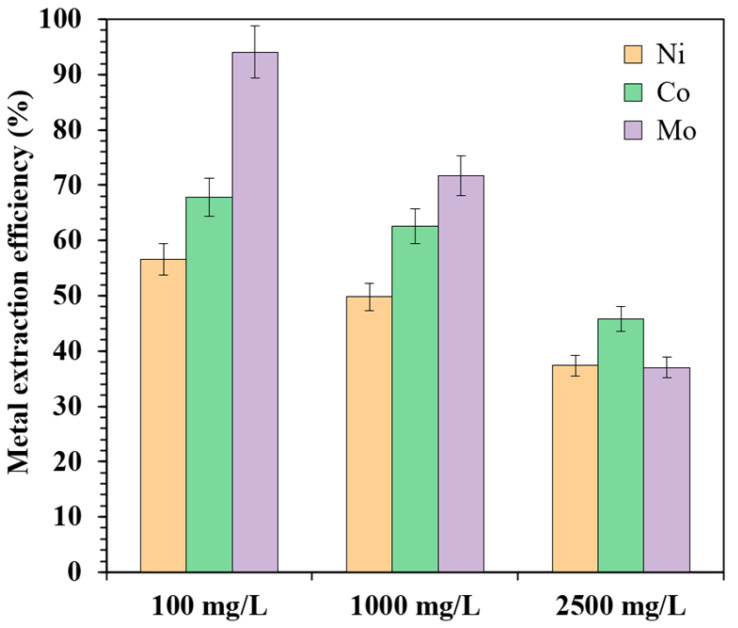
The effect of the metal concentration in the aqueous phase on the Mo, Co, and Ni extraction efficiency.

Figure 3 illustrates the extraction efficiency of the ionic liquid [TOA][Cy272] under varying metal concentrations in synthetic solutions. In these experiments, the organic phase composition was maintained at 35 vol% IL (diluted in 65 vol% kerosene), with an A/O phase ratio of 1. Increasing the metal concentration in the feed solution from 100 mg/L to 2500 mg/L led to a significant decrease in the extraction efficiency for all the investigated metals. Specifically, Mo extraction dropped from 94.1% to 37.0%, Ni from 56.6% to 37.4%, and Co from 67.8% to 45.8%. This decline can be attributed to the limited loading capacity of the [TOA][Cy272] IL. Once the available active sites in the IL phase are saturated, further increases in the metal concentration cannot be accommodated, resulting in reduced extraction efficiency.

In addition to the decline in Mo extraction, the selectivity of the system also deteriorated. The separation factors, βMo/Ni and βMo/Co, decreased sharply from 12.1 to 0.98 and from 7.5 to 0.7, respectively. This indicates that Mo was no longer preferentially extracted over Ni and Co at higher feed concentrations. Such a loss of selectivity at elevated total metal concentrations (e.g., 2.5 g/L) can be primarily attributed to the saturation of the active binding sites in the ionic liquid phase. Once these sites become fully occupied, the extraction process shifts from a selective regime toward non-selective bulk extraction, thereby reducing the preferential uptake of molybdenum over cobalt and nickel [44]. In addition, high metal loadings may promote the formation of aggregated species in the organic phase, such as reverse micelles or metal–extractant clusters, which can enhance the co-extraction of cationic species like Co^2+^ and Ni^2+^. To mitigate these effects and maintain high molybdenum selectivity and extraction efficiency, the feed concentration was optimized at 1000 mg/L in all of the subsequent experiments.

**Figure 4:**
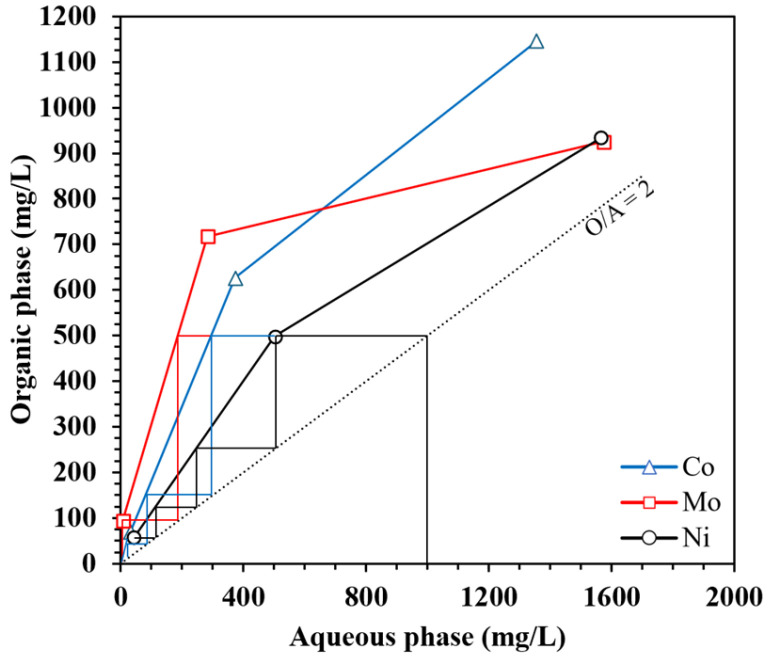
McCabe–Thiele diagram for Mo, Co, and Ni (IL: [TOA][Cy272], O/A: 2, IL concentration of 35%, T: 25 °C).

To better understand the extraction behavior and selectivity of the ionic liquid toward Mo in the presence of Ni and Co, extraction isotherms were constructed using equilibrium data obtained at an organic-to-aqueous phase ratio of 1. Figure 4 shows the equilibrium concentrations of the metals in both phases. Mo exhibited markedly higher extraction efficiency than Ni and Co, particularly at lower metal concentrations. Under these conditions, the extraction efficiencies of Ni and Co ranged from 56.6% to 37.4% and from 67.8% to 45.8%, respectively, confirming the strong selectivity of the ionic liquid for Mo.

A McCabe–Thiele diagram (Figure 4) was constructed using the experimental data. The concentration of each metal in the initial aqueous solution was fixed at 1 g/L, representing an average value relevant to a range of industrial applications. At an O/A ratio of 1, a considerable amount of each metal was extracted in a single stage; however, the operating line lies outside the McCabe–Thiele diagram, indicating that such extraction levels are insufficient to achieve near-complete recovery in a counter-current configuration using [TOA][Cy272] under these conditions. Increasing the O/A ratio to 2 shifts the operating line into the diagram, making multi-stage extraction feasible. Theoretical stage analysis shows that approximately two stages for Mo, three for Co, and four for Ni are needed to reach low residual concentrations in the aqueous phase. The notably lower stage requirement for Mo reflects its higher extraction affinity, making its selective recovery more straightforward. In contrast, the co-extraction of Ni and Co, which requires more stages, adds complexity to process selectivity. Selecting an O/A ratio of 2 provides a balanced approach, optimizing solvent usage, while ensuring effective and selective recovery of molybdenum. Additionally, it aids in identifying methods to minimize the co-extraction of nickel and cobalt.

#### 3.2.3. Effect of IL Concentration

**Figure 5:**
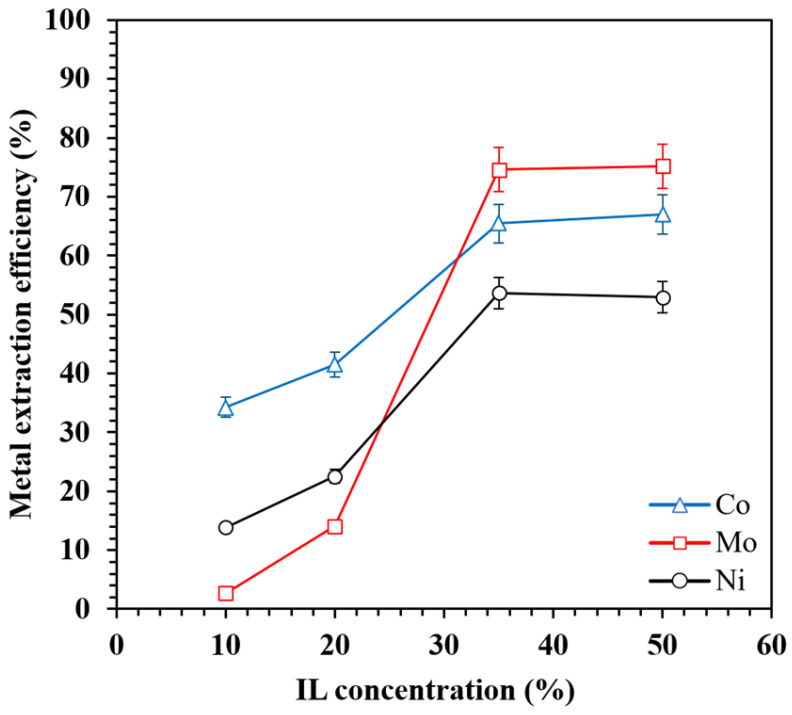
The effect of the [TOA][Cy272] IL concentration on the Mo, Co, and Ni extraction efficiency.

The effect of the [TOA][Cy272] IL concentration on the extraction efficiency of Mo, Ni, and Co was examined by varying the IL content in the organic phase from 10 to 50 vol% (Figure 5). The results show that increasing the IL concentration from 10% to 35% led to a marked improvement in the extraction efficiency for all three metals, with Mo rising from 2.6% to 74.6%, Ni from 13.8% to 53.7%, and Co from 34.3% to 65.4%. This enhancement can be attributed to the greater availability of active extractant molecules in the organic phase, which facilitates complex formation with metal ions at the aqueous–organic interface.

However, beyond 35% IL, no significant improvement in extraction was observed, suggesting that the system approached saturation under the tested conditions. This plateau is commonly associated with the increased viscosity of the organic phase at higher IL concentrations, which impedes mass transfer and reduces interfacial contact efficiency [45,46]. It should be noted that excessive viscosity not only slows diffusion, but can also hinder phase separation and operational handling in practical applications [47]. The selected IL concentration, consisting of 35% by volume of [TOA][Cy272] in kerosene, provided a suitable balance between extraction performance and fluid behavior. The high extraction efficiency observed under the applied conditions, along with the rapid phase disengagement after mixing, suggests that the viscosity of the organic phase remained within a range appropriate for effective liquid–liquid extraction.

At lower IL concentrations, the extraction selectivity followed the order Co > Ni > Mo, indicating limited selectivity for Mo. This trend is likely due to stronger interactions between the [Cy272]^−^ anion and the divalent cations Co^2+^ and Ni^2+^ or their aqueous complexes. When extractant availability is limited, these stronger interactions lead to competitive binding, reducing the extraction of Mo. Molybdenum, typically present as oxoanionic species, such as MoO_4_^2−^, can also exist in polymeric anionic forms (e.g., Mo_7_O_24_^6−^) [48], which may interact less efficiently with the ionic liquid components, further limiting its extraction. In contrast, increasing the IL concentration significantly enhances Mo extraction and shifts selectivity toward Mo over Co and Ni. This behavior suggests that higher extractant levels promote more effective interactions between anionic Mo species and the quaternary ammonium cation ([TOA]^+^), thereby facilitating their extraction.

In conclusion, increasing the IL concentration enhances both the extraction efficiency and Mo selectivity up to an optimal level. However, excessively high IL loadings may be neither economically viable nor operationally practical, due to increased viscosity and diminishing returns in regard to performance. Therefore, an IL concentration of around 35% appears to offer a favorable balance between extraction performance and process efficiency.

#### 3.2.4. Effect of A/O Phase Ratio

**Figure 6:**
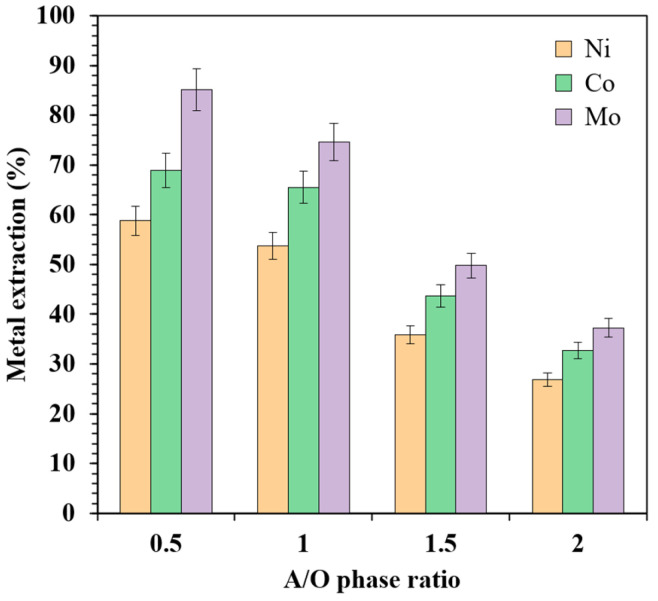
The effect of the A/O phase ratio on the Mo, Co, and Ni extraction efficiency.

The influence of the A/O phase ratio on the extraction efficiency of Mo, Co, and Ni was investigated in the range of 0.5 to 2.0. In all of the experiments, the organic phase consisted of [TOA][Cy272] IL diluted with kerosene at a fixed ratio of 35:65 (*v*/*v*), and the contact time was maintained at 30 min. The results are presented in Figure 6.

As shown, increasing the A/O phase ratio led to a pronounced decrease in the extraction efficiencies of all three metals. Specifically, when the A/O ratio was increased from 0.5 to 2.0, the extraction of Mo decreased sharply from 85.1% to 37.2%, Co from 68.9% to 32.8%, and Ni from 58.8% to 32.8%. This declining trend can be attributed primarily to the reduced volume of the organic phase available per unit volume of the aqueous phase, which in turn limits the total amount of extractant accessible for metal uptake. Given that the extractant has a finite loading capacity, a lower volume of organic phase restricts the maximum quantity of metals that can be transferred from the aqueous phase.

In addition to the limitation imposed by the extractant capacity, a higher A/O ratio may also impair the phase mixing efficiency, particularly in systems without active agitation, which can further hinder mass transfer. These findings highlight the importance of carefully optimizing the phase ratio in practical applications, as it directly affects extraction performance and the overall efficiency of the separation process.

#### 3.2.5. Effect of pH

**Figure 7:**
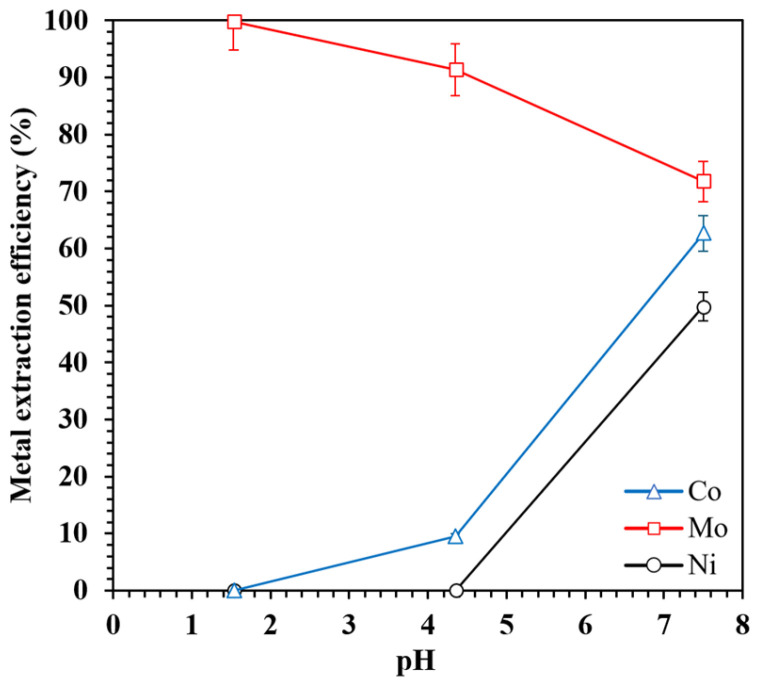
The effect of pH on the Mo, Co, and Ni extraction efficiency.

The pH of the aqueous phase plays a pivotal role in governing both the speciation and solubility of metal ions, as well as the extraction behavior of these ions of extractants. The influence of pH on the extraction efficiency of Mo, Ni, and Co by the [TOA][Cy272] IL was systematically evaluated across a pH range of 1.5 to 7.5 (Figure 7). Higher pH values (above 7.5) were not considered due to the onset of precipitation and the formation of solid hydroxide species [49]. Operational conditions, including an organic phase consisting of 35 vol% of ionic liquid (diluted in 65 vol% kerosene), an A/O phase ratio of 1, room temperature, a stirring rate of 250 rpm, and an extraction time of 30 min, were kept constant to ensure consistency across all of the experiments.

The experimental results revealed a clear dependence of metal extraction on the pH. As the pH increased, the extraction efficiency of Co rose from approximately 0% to 62.6%, and Ni from ~0% to 49.8%. In contrast, Mo extraction decreased from 99.8% at a low pH to 71.8% at a higher pH. This opposing trend can be attributed to the metals’ pH-dependent aqueous speciation and differing extraction mechanisms. Molybdenum exists predominantly as anionic species (e.g., MoO_4_^2−^ or HMoO_4_^−^) under acidic to near-neutral conditions, and is selectively extracted via ion-pair formation with the quaternary ammonium cation [TOA]^+^. The [Cy272]^−^ anion remains in the organic phase to maintain charge neutrality and stabilize the organic environment. At a higher pH, increased competition from hydroxide ions and a shift in Mo speciation likely reduce the efficiency of this ion-pair extraction pathway.

In contrast, Ni^2+^ and Co^2+^ are present as cationic species in aqueous solution, and their extraction improves at moderately acidic to near-neutral pH values. This enhancement is attributed to the deprotonation of [Cy272]^−^, which enables it to act as a coordinating ligand and form neutral metal–ligand complexes with Co^2+^ and Ni^2+^ [50]. The reduced proton competition at a higher pH further favors these interactions, facilitating the transfer of Co and Ni into the organic phase. Thus, the bifunctional nature of the ionic liquid enables the selective extraction of Mo at a low pH and the increased extraction of Co and Ni at a higher pH, driven by distinct mechanistic pathways.

These findings demonstrate that [TOA][Cy272] exhibits strong selectivity for Mo in highly acidic media, enabling its effective separation from Ni and Co under such conditions. This pH-dependent selectivity suggests that staged or sequential extraction processes based on controlled pH adjustment could efficiently separate and recover Mo from metal mixtures containing base metals. Moreover, the fixed A/O phase volume ratio of 1 used throughout this study provided balanced phase volumes, facilitating effective phase separation and consistent mass transfer. The high selectivity observed under these conditions indicates that variation of the A/O ratio is unlikely to further improve the selectivity within the tested system.

### 3.3. Stripping

To recover Mo from the loaded [TOA][Cy272] IL and transfer it into the aqueous phase, two stripping agents, hydrochloric acid and ammonium hydroxide, were evaluated. While selective extraction is critical for the purification step, the stripping stage is equally important, as it determines the overall separation efficiency and selectivity [51]. The organic phase used in the stripping experiments was obtained from the extraction stage, where a solution containing 1 g/L each of Mo, Co, and Ni at pH 7 was used. The extraction was performed under conditions of 35 vol% of IL, an A/O phase ratio of 1, and a contact time of 30 min. This approach was chosen to ensure that all the metals (Mo, Co, and Ni) were present in the organic phase, eliminating any influence of selectivity that might arise if a selectively extracted organic phase was used. Under these conditions, the loaded IL phase contained approximately 690 mg/L of Mo, 660 mg/L of Co, and 503 mg/L of Ni. Notably, the concentrations of the studied metals in the loaded organic phase were nearly identical, which is essential for a comprehensive evaluation of the stripping efficiency.

**Figure 8:**
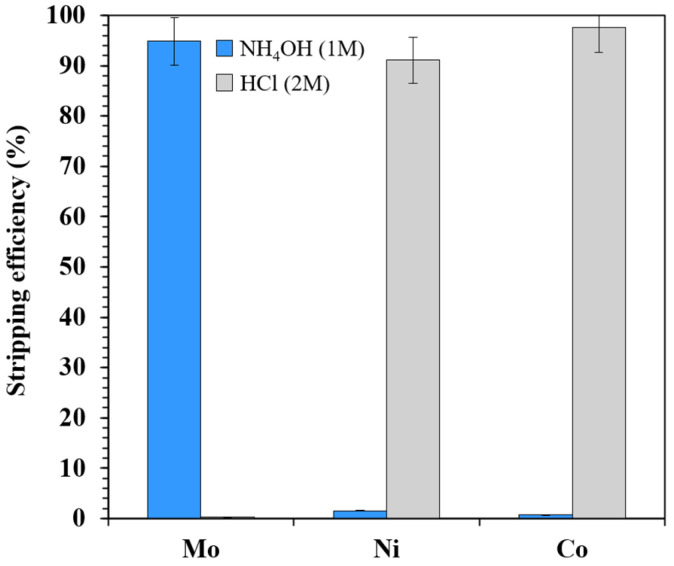
[TOA][Cy272] IL stripping efficiency using HCl and NH_4_OH.

Figure 8 presents the stripping efficiencies using 2 M HCl and 1 M NH_4_OH at room temperature, with an A/O phase ratio of 1. The results show that HCl was ineffective in stripping Mo, but efficiently stripped Ni (92%) and Co (98%). In contrast, NH_4_OH exhibited outstanding selectivity for Mo, achieving a stripping efficiency of ~95%, while Co and Ni were barely stripped (below 1.5%).

This behavior can be attributed to differences in metal speciation and interactions with the IL under acidic and alkaline conditions. Under alkaline conditions, Mo (present as anionic species such as MoO_4_^2−^) forms soluble molybdate complexes that readily transfer into the aqueous phase. Meanwhile, Co and Ni either remain strongly associated with the IL anion ([Cy272]^−^) or form insoluble hydroxides, leading to poor stripping. Conversely, under acidic conditions, the protonation of [Cy272]^−^ facilitates the release of divalent cations like Ni^2+^ and Co^2+^, while Mo remains retained due to the stability of the complex.

These results highlight the importance of selecting a stripping agent according to the target metal. HCl is highly effective for Co or Ni recovery, whereas NH_4_OH offers a selective and efficient route for Mo recovery. This dual-stripping strategy enhances the operational flexibility of the [TOA][Cy272]-based system for selective metal separation from complex aqueous mixtures.

Based on these results, the overall recovery efficiency of Mo via the combined extraction and stripping process under optimal conditions reached approximately 95%, while Co and Ni recoveries remained negligible. This demonstrates the strong potential of [TOA][Cy272] as a selective ionic liquid for Mo recovery from multicomponent systems. Although HCl showed excellent stripping efficiency for both Co and Ni, its use also resulted in the loss of Mo selectivity, and it is not recommended in applications targeting Mo recovery. Moreover, in the case of NH_4_OH, the concentrations of Ni and Co in the stripped solution were below 7 mg/L, which is too low to justify further recovery under the tested conditions. As the main objective of this study was the selective extraction and recovery of Mo, the recovery and treatment of residual Co and Ni were not further investigated. Nevertheless, strategies such as sulfide or oxalate precipitation for base metal recovery, as well as the treatment of residual metal ions in the aqueous phase, represent relevant directions for future research aimed at full resource recovery and effluent minimization.

## 4. Conclusions

The bifunctional ionic liquid [TOA][Cy272] proved to be highly effective for the selective recovery of molybdenum from mixed Co–Ni–Mo solutions. Under optimal conditions (35 vol% IL, pH of 2, and 30 min of contact time), more than 90% of Mo was extracted. At the same time, Co and Ni remained mainly in the raffinate, confirming that the IL reverses the parent extractant’s Co selectivity in favor of molybdate anions. The extraction efficiency rose with the IL concentration up to 35 vol%, beyond which the increased viscosity curtailed further gains, and high metal loadings (>1 g/L) shifted the system from selective to bulk extraction as the IL became saturated.

Selective stripping completed the separation: 1 M NH_4_OH stripped approximately 95% of the loaded Mo, with negligible co-stripping of Co or Ni, whereas 2 M HCl quantitatively recovered Co and Ni, while leaving Mo in the organic phase. Integrating one extraction and one stripping stage, thus, delivered an overall Mo yield of roughly 95% and enabled straightforward IL regeneration. Synthesized from inexpensive, commercially available reagents and operating under mild, non-volatile conditions, [TOA][Cy272] offers a green and economically attractive route for valorizing spent hydrodesulfurization catalysts and related secondary resources. Future work should focus on continuous counter-current operation, IL stability and loss minimization, the treatment of real leach liquors containing additional impurities, and comprehensive techno-economic and life-cycle analyses to benchmark the process against conventional solvent–extraction circuits. In addition, future studies will investigate the recyclability and long-term stability of [TOA][CY272] through multi-cycle extraction–stripping experiments to further assess its industrial applicability.

## Figures and Tables

**Table 1:** Extraction efficiencies of TOA, Cyanex 272, and [TOA][Cy272] IL for Mo, Co, and Ni.

Component	Extraction (%)			Selectivity		
	Mo	Ni	Co	βMo/Ni	βMo/Co	βCo/Ni
TOA	~0.0	~0.0	~0.0	-	-	-
Cyanex 272	86.3 ± 4.3	12.1 ± 0.5	98.5 ± 4.1	45.65	0.01	475.73
[TOA][Cy272] IL	71.8 ± 3.2	49.8 ± 2.5	62.6 ± 3.3	2.58	1.53	1.69

## Data Availability

The original contributions presented in this study are included in the article material. Further inquiries can be directed to the corresponding author(s).
